# Climate and habitat configuration limit range expansion and patterns of dispersal in a non‐native lizard

**DOI:** 10.1002/ece3.7284

**Published:** 2021-02-22

**Authors:** Robert J. Williams, Alison M. Dunn, Lily Mendes da Costa, Christopher Hassall

**Affiliations:** ^1^ Faculty of Biological Sciences School of Biology University of Leeds Leeds LS2 9JT UK

**Keywords:** climate matching, heterogeneous landscape, invasive species, lag phase, *P. muralis*, range expansion

## Abstract

Invasive species are one of the main causes of biodiversity loss worldwide. As introduced, populations increase in abundance and geographical range, so does the potential for negative impacts on native communities. As such, there is a need to better understand the processes driving range expansion as species become established in recipient landscapes. Through an investigation into capacity for population growth and range expansion of introduced populations of a non‐native lizard (*Podarcis muralis*), we aimed to demonstrate how multi‐scale factors influence spatial spread, population growth, and invasion potential in introduced species. We collated location records of *P. muralis* presence in England, UK through data collected from field surveys and a citizen science campaign. We used these data as input for presence‐background models to predict areas of climate suitability at a national‐scale (5 km resolution), and fine‐scale habitat suitability at the local scale (2 m resolution). We then integrated local models into an individual‐based modeling platform to simulate population dynamics and forecast range expansion for 10 populations in heterogeneous landscapes. National‐scale models indicated climate suitability has restricted the species to the southern parts of the UK, primarily by a latitudinal cline in overwintering conditions. Patterns of population growth and range expansion were related to differences in local landscape configuration and heterogeneity. Growth curves suggest populations could be in the early stages of exponential growth. However, annual rates of range expansion are predicted to be low (5–16 m). We conclude that extensive nationwide range expansion through secondary introduction is likely to be restricted by currently unsuitable climate beyond southern regions of the UK. However, exponential growth of local populations in habitats providing transport pathways is likely to increase opportunities for regional expansion. The broad habitat niche of *P. muralis*, coupled with configuration of habitat patches in the landscape, allows populations to increase locally with minimal dispersal.

## Graphical abstract

We investigated the potential for population growth and range expansion of introduced populations of a non‐native lizard (*Podarcis muralis*), considering multi‐scale factors influencing growth, and spatial spread. National‐scale models indicated climate suitability restricted to the southern parts of the UK, limited by a latitudinal cline in overwintering conditions. Patterns of local population growth and range expansion were related to differences in local landscape configuration and heterogeneity. The broad habitat niche of *P. muralis*, coupled with configuration of habitat patches in the landscape, allows populations to increase locally with minimal dispersal

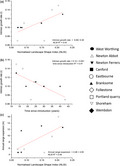

## 1 INTRODUCTION

The global rise in the number of species introduced to regions beyond their native range via human‐mediated translocation shows no sign of reaching saturation point (Seebens et al. 2017). While many species fail to establish or have little negative effect following introduction, a subset of these do spread and can have significant impact on economies, human health, native biodiversity, and ecosystem services (Keller et al. 2011; Kolar & Lodge, 2001; Vila et al. 2010). The severity of potential negative impacts (e.g., local or even global extinction of native species) is such that invasive non‐native species (INNS) are justifiably regarded as one of the most significant threats to biodiversity worldwide (Genovesi, 2009; Simberloff et al. 2013).

For non‐native species to become widespread and potentially damaging following introduction to new regions, introduced populations must negotiate the three stages of an introduction–establishment–invasion continuum (Blackburn et al. 2011). Evaluation of the likelihood of a species to be transported, establish, and to spread, as well as the potential for having ecological, economical, and health impacts, forms the basis of “invasive” risk assessment for alien species (Bacher et al. 2018; Roy et al. 2019). Although it has been argued that the term “invasive” does not always necessarily equate with a species’ negative impact (Ricciardi & Cohen, 2007), the potential for damaging effects inherently increases as introduced species increase in population size and spread across novel landscapes, thus affecting broader areas and more ecological communities (Crooks, 2005). As such, there is great interest in understanding patterns and rates of expansion of introduced species, and the environmental factors which limit their distributions (Gallien et al. 2010; Roy et al. 2019).

Following introduction and successful establishment beyond native ranges, species can further expand their range through local dispersal processes and/or by jump dispersal events that may be human‐mediated (i.e., deliberate or accidental movement of individuals between habitats). Invading species typically exhibit several phases in the rate of spread. Firstly, there is an initial establishment phase where rate of spread is slow, secondly, an expansion phase typified by increasing rates of spread, and finally, a saturation phase when available space is occupied and expansion rates reach a plateau (Arim et al. 2006).

A suite of factors influences patterns and rates of range expansion during these phases: propagule size, dispersal mode, matching of physiological and ecological traits of invading species with environmental conditions at the receptor site, vital rates (births and deaths), species interactions, evolutionary processes, spatial heterogeneity, and temporal variability (Enders et al. 2020). Furthermore, our ability to assess and predict the temporal dynamics of invasions is often complicated by the phenomenon of lag phases; wherein, an introduced species remains at low population levels in the early stages of establishment for a protracted period of time before the sudden onset of rapid range expansion (see Crooks (2005) for review of the causes of temporal lags at all stages in the invasion process). Introduced populations of the northern Raccoon (*Procyon lotor*), for example, remained small for a number of years following introduction to Europe before a population explosion in the mid 1990s (Salgado, 2018). Similarly, landscape complexity can result in temporal and spatial patterns of invasion dynamics that deviate from classic theory of symmetrical, radial expansion from a central point (diffusion theory) (Kinezaki et al. 2010; Shigesada et al. 1995; Skellam, 1951). The effects of landscape heterogeneity on patterns and rates of expansion have been demonstrated in the quick colonization of areas of suitable habitat in the early stages of the American mink (*Neovison vison*) invasion, compared with uptake in areas of low habitat suitability in Scotland (Fraser et al. 2015), and the fluctuating rates in range expansion of Cane toad (*Rhinella marina*) in response to changing environmental conditions in newly invaded areas of Australia (Urban et al. 2008). Consideration of dispersal processes across heterogeneous landscapes is therefore central to predicting potential for range expansion during the invasion process (Bocedi et al. 2014; Grayson & Johnson, 2018; Travis et al. 2011). The development of platforms for spatially explicit individual‐based modeling (Bocedi, Zurell, et al., 2014; Samson et al., 2017) has enabled the nested interactions between dispersal, landscape properties, and population dynamics to be considered in predicting species distributions, increasing the ecological realism of range expansion models (Andrew & Ustin, 2010; Ferrari et al. 2014; Hunter‐Ayad & Hassall, 2020; Mang et al. 2018).

In this study, we determine the potential for range expansion of the non‐native common wall lizard (*Podarcis muralis*) in the UK. *Podarcis muralis* has a long history of introductions beyond its native range which covers most of Western and Southern Europe (Gassert et al. 2013). Many of these introductions continue to extend the species’ range throughout continental Europe (Oskyrko et al. 2020; Šandera, 2017; Santos et al. 2019; Wirga & Majtyka, 2015), but the species also has several populations established in the New World, both in the United States (Brown et al. 1995) and Canada (Allan et al. 2006). Introduced to Vancouver Island, British Columbia, in 1970, the species persisted in isolated populations until 2006, but has since spread with alarming speed due to jump dispersal (human‐mediated) and natural radial dispersal of 40–70 m a year in urban areas (Engelstoft et al. 2020).

To date, there is no empirical evidence of negative ecological impacts of *P. muralis* introductions in the UK, and there is mixed social perception and opinion toward the species’ presence (Williams et al. 2019). However, suspected declines in native lizards through interference and/or exploitation competition have been reported following introductions of *P. muralis*, to both Germany (Kühnis & Schmocker, 2008; Münch, 2001; Schulte, 2009) and the UK (Mole, 2010).

There have been multiple introduction events of *P. muralis* to the UK both as deliberate releases of captive animals and as cargo stowaways, with some extant populations having been established on the UK mainland as early as the 1970s (Michaelides et al. 2013). More recent introductions (1980s onward) have mostly arisen from movement of individuals from already established populations (secondary introduction) or captive‐bred animals, rather than directly sourced from the native range (Michaelides et al. 2015). The UK populations represent the species at the northern extent of its range, with sites having markedly different climatic conditions compared to source regions of the native range. For example, air temperatures experienced by populations during the main activity season at sites in England are 5–10°C lower than their source regions in Tuscany and western France (While et al. 2015).

We investigated the potential for range expansion of *P. muralis* in the UK with models highlighting different (but complementary) parameters likely to influence spread at two spatial scales. Firstly, since long‐distance jump dispersal via translocation is important in facilitating spread of this species, we aimed to predict the national extent of the area potentially available for further colonization by running a species distribution model (SDM) based on current climatic suitability at these northern extremes. As has been speculated elsewhere, the ability to survive cold winters is likely limiting to the spread of introduced *Podarcis* populations (Burke et al. 2002). We, therefore, hypothesized that latitudinal clines in climate would restrict the area available for northward expansion of *P. muralis* via long‐distance human‐assisted translocation in the UK. Climate change scenarios may significantly affect the future extent of habitat suitability for *P. muralis*. However, in this study, we are primarily interested in trying to understand what is limiting the current distribution and not necessarily trying to predict where the species’ will be in the future.

Second, to make predictions of population growth and dispersal patterns, as well as identify environmental features important to range expansion at a local level, we took a hybrid model approach combining SDMs, informed by variables characterizing 10 local landscapes (i.e., microclimate, proximity to geographic features, and habitat type), with a high resolution (15 × 15 m) spatiotemporal individual‐based model (IBM) simulating local population and dispersal dynamics. We expected that landscape characteristics (i.e., configuration and connectivity of suitable habitat patches) would result in asymmetrical patterns of predicted dispersal within populations, which in turn would result in spatial and temporal variance in patterns of population growth and range expansion between populations. These analyses allow us to investigate the proximate and ultimate barriers to spread, as well as simulating the potential for invasion lag in each population.

## 2 METHODS

**FIGURE 1:**
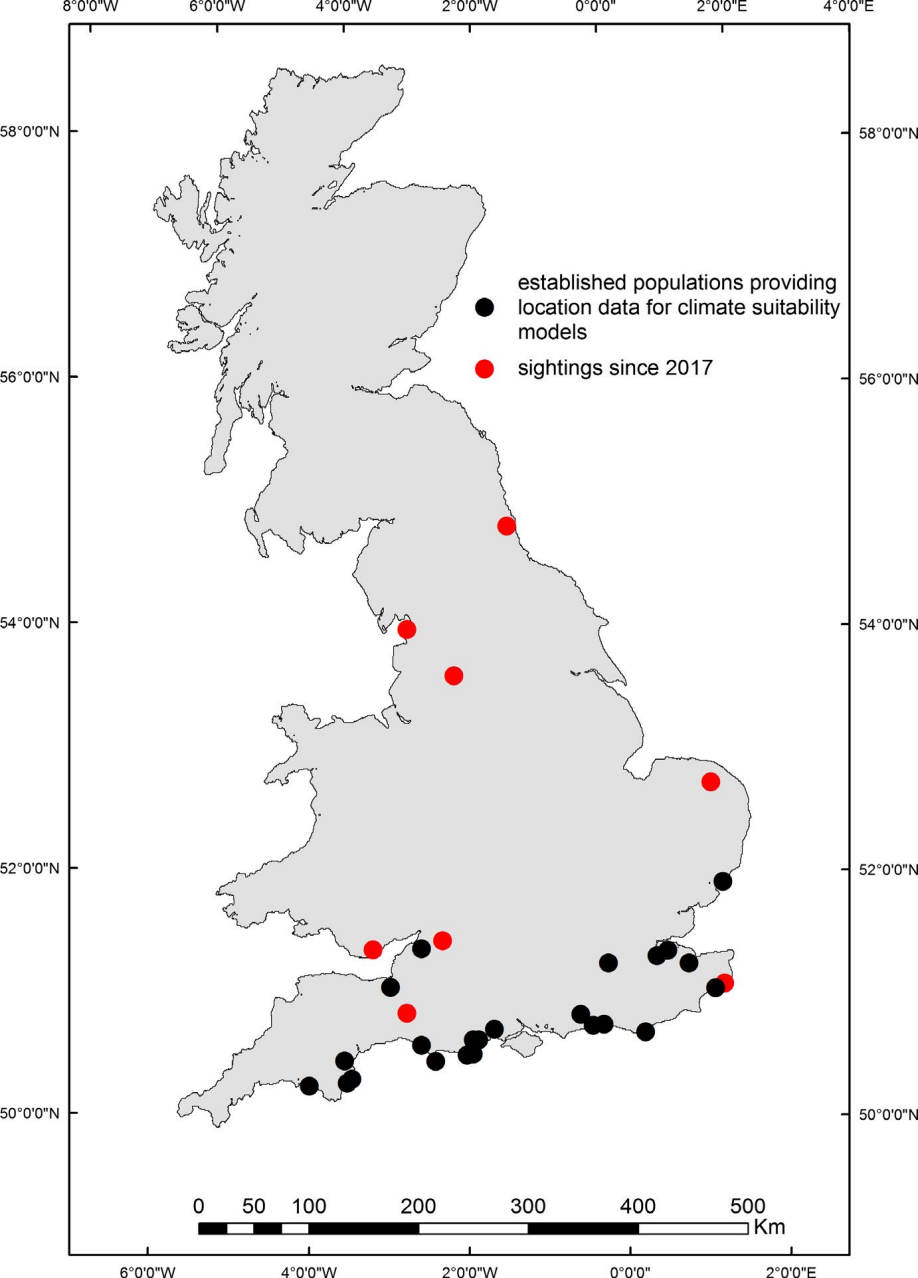
Locations of UK breeding populations of *Podarcis muralis* (black) from which presence data informed models of climatic suitability (*n* = 25) and locations of confirmed sightings (red) arising from a citizen science campaign 2017–2019 (*n* = 8)

The locations of known established *P. muralis* populations were obtained from data collated on the Wall Lizard Project hosted on the Surrey Amphibian and Reptile Group website (Langham, 2019). We determined the current geographic extent of as many of these populations as logistically possible (10 sites) using a combination of visual surveys, canvassing of the local public at sites of interest, and press releases in local and regional media encouraging members of the public to report their wall lizard sightings (see Appendix S1). Of the 30 extant populations recorded on the UK mainland, we visited 21 between three field seasons (April–September) of 2016, 2017, and 2018. We did not visit the remaining nine locations because the lizard populations were either known to be very small, had no recent confirmed sightings and no accurate location data, access was restricted, and/or site locations were otherwise logistically challenging (i.e., distance from other populations). Specific attention was given to assessing the extent of *P. muralis* presence along railway habitat at West Worthing, Sussex (50.818°N, 0.390°W) during a five‐week period in June–July 2018—the railway acting as a linear transect, along which we could assess the utility of railway habitat as a corridor for dispersal (Figure S1 Appendix S1). We also collected wall lizard location data from populations at two additional sites, Eastbourne (50.768°N, 0.291°E) and Kingswear (50.349°N, 3.568°W). The former was confirmed to be an established colony through a site visit, and the latter was reported to RW by a member of the public responding to a citizen science campaign in regional media. Sightings were confirmed for a further eight new locations as a result of the citizen science campaign (Figure 1) (see Appendix S1 for detailed methods). Overall, we collated a total of 1,331 lizard sightings (76 from online portal, 52 from postcard returns, and 1,203 from visual surveys) across 25 sites (Figure 1).

### 2.1 Modeling climate suitability across the UK

We used our UK *P. muralis* presence data and records from the native range (GBIF.org, 2020) to develop relative habitat suitability maps at the UK national extent using MaxEnt v3.3.3k software (Phillips et al. 2006). We minimized spatial autocorrelation (due to possible clustered sampling effort) between data points by thinning all data to a minimal distance of 10 km between points using the R package SpThin (Aiello‐Lammens et al. 2015), resulting in 25 UK records and 1,542 from the native range to be used in the models. *Podarcis muralis* has demonstrated rapid adaptive responses following introduction to cool climates, with ability to evolve broader thermal tolerance at dispersal fronts (Litmer & Murray, 2019), and prolonged embryo retention and faster embryonic growth at low temperatures—compared with ancestral states (While et al. 2015). To best reflect the current climatic tolerance of the species, we therefore compared models that used *P. muralis* presence records from the introduced UK range only (acknowledging the possibility for regional overfitting with such data), with models constructed using only native occurrence data, and the UK and native occurrence data combined.

### 2.2 Environmental variables

We selected 10 climatic variables that have most relevance to wall lizard biology and therefore likely to influence distribution (Wirga & Majtyka, 2015) (Table 1), obtained from WorldClim—Global Climate Data (Fick & Hijmans, 2017) and E‐OBS datasets from the EU‐FP6 project ENSEMBLES (Cornes et al. 2018). Due to the different resolution in data from these sources, we up‐scaled E‐OBS climatic variables by using the bilinear interpolation to a spatial resolution of 0.0083°. These 10 variables were refined from an initial input of 13 climate variables through an iterative process of removal/retention to limit covariate correlation (Spearman's rank correlation; correlated if *r*_s_ ≥ 0.6) and maximize model performance (Glover‐Kapfer, 2015). We kept parameter settings in MaxEnt the same for modeling at the national and local levels (see Appendix S1 for detailed method).

**TABLE 1:** Details of variables and their data source used in MaxEnt models of habitat suitability for *Podarcis muralis*

Model	Environmental variable	Description	Resolution	Source
National	Winter min/max temp	Average of monthly mean min and max temp °C 1970–2000	0.0083°	WorldClim data
	Summer min/max temp	Average of monthly mean min and max temp °C 1970–2000	0.0083°	WorldClim data
	Spring solar radiation	Average of spring mean max solar radiation (kJ/m^2^ day^−1^) 1970–2000	0.0083°	WorldClim data
	Autumn growing season	Average number of autumn days where daily mean temperature > 5°C	0.25°	E‐OBS data
	Spring growing season	Average number of spring days where daily mean temperature > 5°C	0.25°	E‐OBS data
	Frost days of spring	Average number of spring days where daily minimum temperature < 0°C	0.25°	E‐OBS data
	Summer days of summer	Average number of summer days where daily maximum temperature > 25°C	0.25°	E‐OBS data
	Ice days of winter	Average number of winter days where daily minimum temperature < 0°C	0.25°	E‐OBS data
Local	NDVI	Normalized difference vegetation index	2m	Calculated from Landsat 8 OLI/TIRS (USGS, 2017))
	Distance to nearest buildings	Euclidian distance to buildings	2m	Calculated from OS Open Map (1:10,000) (EDINA, 2018)
	Distance to nearest roads	Euclidian distance to roads	2m	Calculated from OS Open Map (1:10,000) (EDINA, 2018)
	Distance to rail	Euclidian distance to railway tracks	2m	Calculated from OS Open Map (1:10,000) (EDINA, 2018)
	Spring insolation	Mean incoming solar insolation 1981–2017	2m	Calculated in ArcMap from Lidar DSM 2m (Environment Agency, 2017)
	Phase 1 habitat	Habitat classification	2m	

### 2.3 Modeling local habitat suitability

A total of 1,083 presence records (all direct observations during visual surveys), across 10 study locations representing the range of habitats used by *P. muralis* (urban, suburban, and rural), were used in producing relative habitat suitability maps and predictive models of local range expansion. These study sites encompassed heterogeneous land cover that helped in identifying variables affecting local habitat suitability and features acting as important corridors for range expansion. Data for six environmental variables at 2 m resolution were used for the MaxEnt input and are summarized in Table 1. All variables were calculated and prepared in ArcGIS^®^ (Esri 2017). We used the Phase One Habitat Survey Toolkit (Centre for Ecology Environment & Conservation, 2018) to create fine‐scale habitat type (categorical) data layers.

Sampling bias is often inherent in occurrence data (Merow et al. 2013) and there are several aspects of our approach to modeling habitat suitability at the local level which cumulatively addresses any issue of a bias effect in our presence data: (a) The points of local introduction are explicitly known for the various populations studied, and thus, clustered occurrence data are inevitable (and expected). (b) Our combined survey methods (citizen science, canvasing, and visual surveys) ensured we could confidently survey from the core of the population out toward the extremities, giving confidence to our assessment of habitat use and local range extent. (c) Our models use as input occurrence data and manually generated background points from across all populations/study sites. The model was then projected to each of the study sites separately. Local projections, therefore, represented the suite of habitat types used (ranging from rural to highly urbanized) across all sites—offsetting any local clustering around features (i.e., buildings and roads).

### 2.4 Modeling local range expansion (IBM)

We used spatially explicit individual‐based models to identify the functional responses of *P. muralis* to the habitat structure provided by MaxEnt models. These models were constructed using the platform RangeShifter v1.1 (Bocedi, Palmer, et al., 2014) where behaviors of individuals within a population are simulated in relation to life‐history parameters and conditions determined by a set of spatial inputs. The local habitat suitability maps generated by MaxEnt were the basis of the spatial inputs used for individual‐based models and included: habitat quality landscape layers that were created by rescaling the MaxEnt logistic values (estimates between 0 and 1 of relative suitability) to a scale of 1–100 to represent the percentage of maximum carrying capacity that a cell can support; and cost surface layers (where cell values represent the resistance for dispersing individuals to move through cells), created based on a reciprocal transformation of habitat suitability resulting in a scale of matrix hostility of 1–10 (Hunter‐Ayad & Hassall, 2020) (Figure S2 Appendix S1). All inputs were resampled using bilinear interpolation to 15 × 15 m cell size to reduce demands on computational memory while retaining relevance to wall lizard movement capabilities. A single cell in each landscape was identified as the initial species distribution (i.e., point of introduction for each population, respectively) based on knowledge of the precise location of introduction when known, or by using the center point of the current extent of sighting records for the population.

### 2.5 Parameterization

Parameters of wall lizard demographics and behavioral attributes were based on empirical data in the published literature. Parameterization was further calibrated through an iterative process, where simulations were repeated across all study sites with fine parameter adjustments within biologically meaningful limits until a single set of parameters was found that modeled as closely as possible the currently observed spatial extent of each study population (Fraser et al. 2015). Where published empirical data were not available, reasonable judgments and/or simplifying assumptions were made that were biologically realistic and justifiably reflect the functional biology of *P. muralis* (Table S1 in Appendix S1).

### 2.6 Initialization

Simulations were initialized using known founder size where documented (Langham, 2019; Michaelides et al. 2015). Where founder size was unknown, we used a minimal founder size that resulted in reasonable simulation outputs as per the iterative process mentioned above. We assumed adult age class for all founders. Local extinction probability (probability every population (cell) has in each year of going extinct) was set at a constant of 0.003 across study sites (derived by the iterative process mentioned above) to add an element of stochasticity to the model. Simulations (50 replicates) of population range expansion for the 10 study populations were then run for the period of time since introduction (which varies among sites) up to the year 2040.

## 3 ANALYSIS

We investigated how landscape characteristics might influence population size, rate of population growth, and range expansion by first obtaining standard population growth metrics: carrying capacity (*K*), and intrinsic rate of increase (*r*), from linear growth curves applied to mean yearly population size data taken across all simulation iterations in R (R Core Team, 2017) using the package growthcurver (Wagner, 2016). We then created binary habitat suitability layers, depicting suitable and unsuitable cells from our MaxEnt outputs for a radius of 200 m around introduction points. We used the maximum test sensitivity plus specificity logistic threshold (that which the MaxEnt models maximize their discrimination of presences from background data (Glover‐Kapfer, 2015; Jimenez‐Valverde & Lobo, 2007; Merow et al. 2013)) to delineate suitable versus unsuitable cells. Binary layers then served as inputs for spatial analysis of suitable patch configuration in the program FRAGSTATS v4 (McGarigal et al. 2002). An illustration of workflow in layer preparation from SDM to IBM to FRAGSTATS input is provided in Figure S2 Appendix S1.

We ran (simple stepwise) linear regression models with two FRAGSTAT metrics describing heterogeneity of suitable habitat patches within the landscape (Normalized Landscape Shape Index—a measure of patch aggregation where NLSI = 0 when the landscape consists of a maximally compact patch of the corresponding type and increases (to a maximum of 1) as the patch type becomes increasingly disaggregated; and Connectance—a measure of functional joinings of patches reported as a percentage of the maximum possible connectance given the number of patches) and average habitat quality as explanatory variables, and the growth rate parameters (*K*, *r*) and annual range expansion as response variables. We set the threshold distance within which patches are deemed "connected" to an arbitrary 100 m. Values for NLSI and Connectance for each site are provided in Table S2 Appendix S1.

## 4 RESULTS

### 4.1 National‐scale climatic suitability

The MaxEnt model fit at the UK national scale using only presence points from the non‐native range had an average test and training AUC score of 0.99 (*SD* = 0.01). The most important variable to the model was number of summer days, which made the highest relative percent contribution to the model (48.8%) followed by ice days of winter (38.8%); whereas, maximum and minimum winter temperatures made no contribution (0%). Models based solely on presence record from the native range, and native‐UK records combined, had average test and training AUC scores of 0.87 and 0.86, respectively. Spring radiance made the highest relative contribution to both models and minimum winter temperature the least contribution.

**FIGURE 2:**
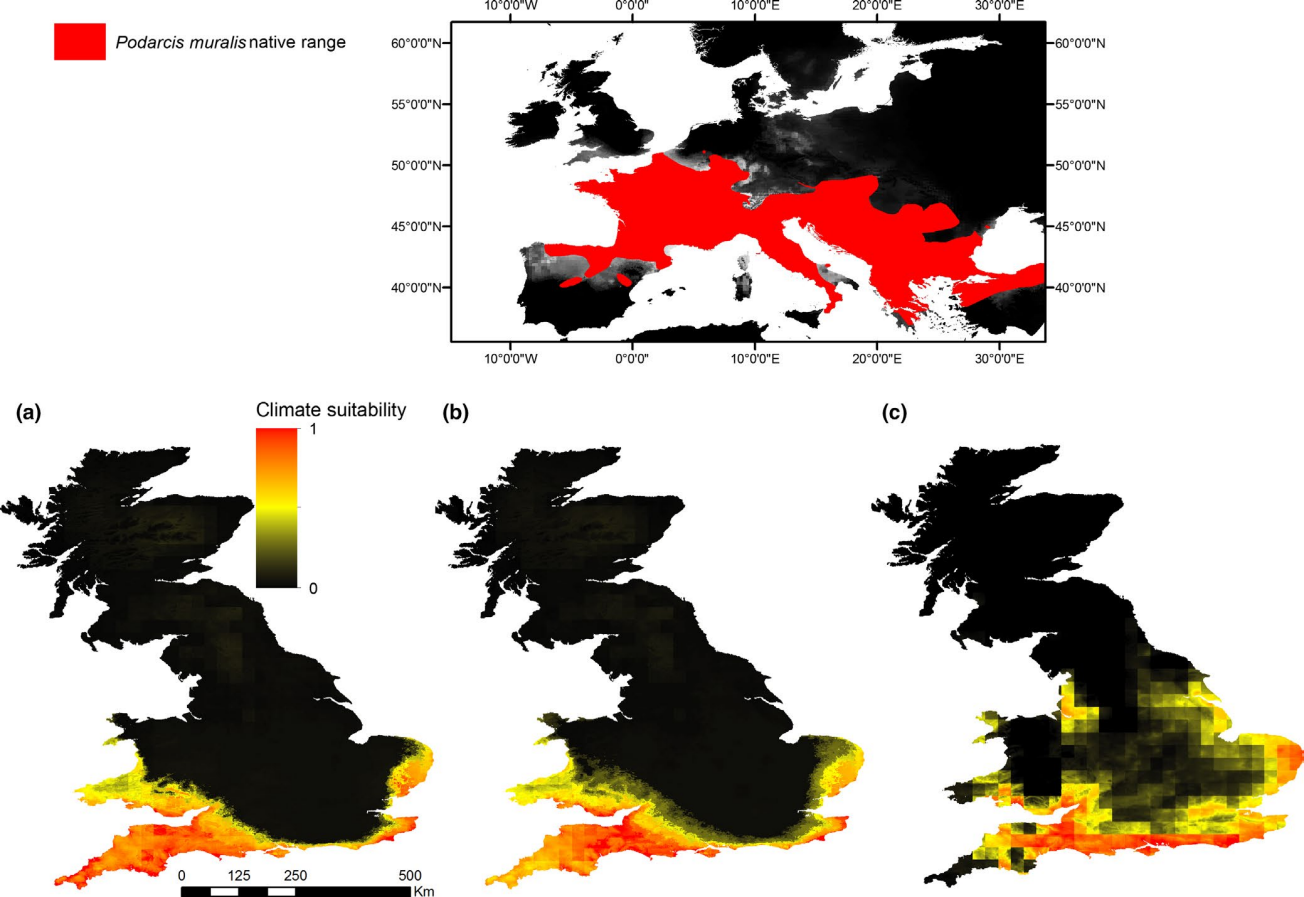
Regions of climatic suitability for *Podarcis muralis* in the UK as predicted by MaxEnt models considering seasonal averages of 10 climate variables and presence data from (a) native range on continental Europe, (b) native range and introduced UK populations, and (c) introduced UK populations only. Map inset shows *P. muralis* native continental range

The models indicate suitable climatic conditions for *P. muralis* in maritime climates all along the south coast of the UK—from Norfolk in the southeast, to the south coast of Wales (Figure 2a–c). Suitable conditions inland diminish toward a latitude of ~52°N, but are particularly evident in the Greater London Metropolitan area when only the UK records were considered (Figure 2c). The pattern of response to climatic variables across models can be generalized as increasing suitability with covariates indicative of milder winters and spring radiance. Response curves for each model are presented in Figure S3 Appendix S2.

### 4.2 Local scale habitat suitability

**FIGURE 3:**
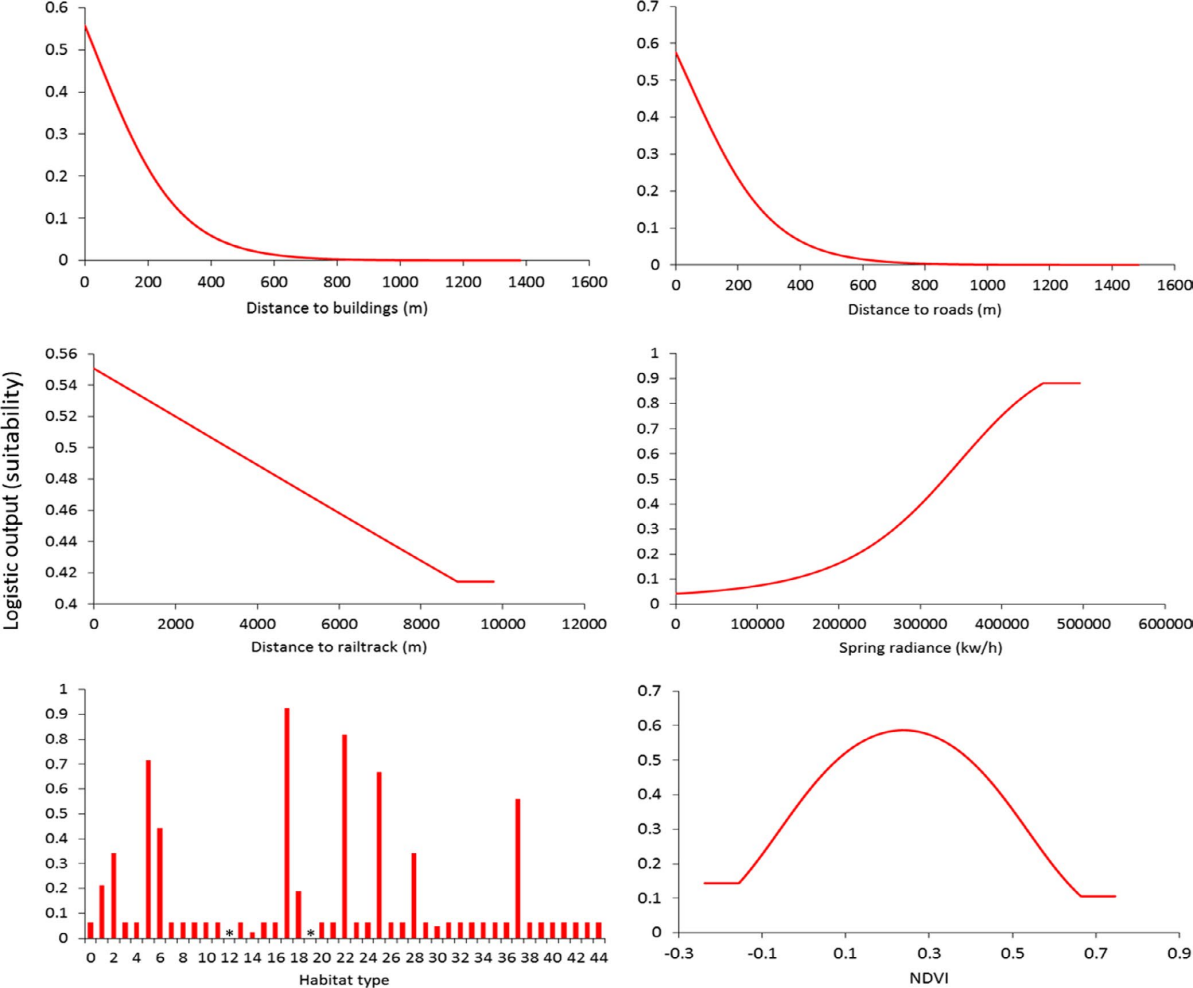
Response curves (habitat suitability) for *Podarcis muralis* to six environmental variables as modeled in MaxEnt considering 10 sites at the UK local scale

**FIGURE 4:**
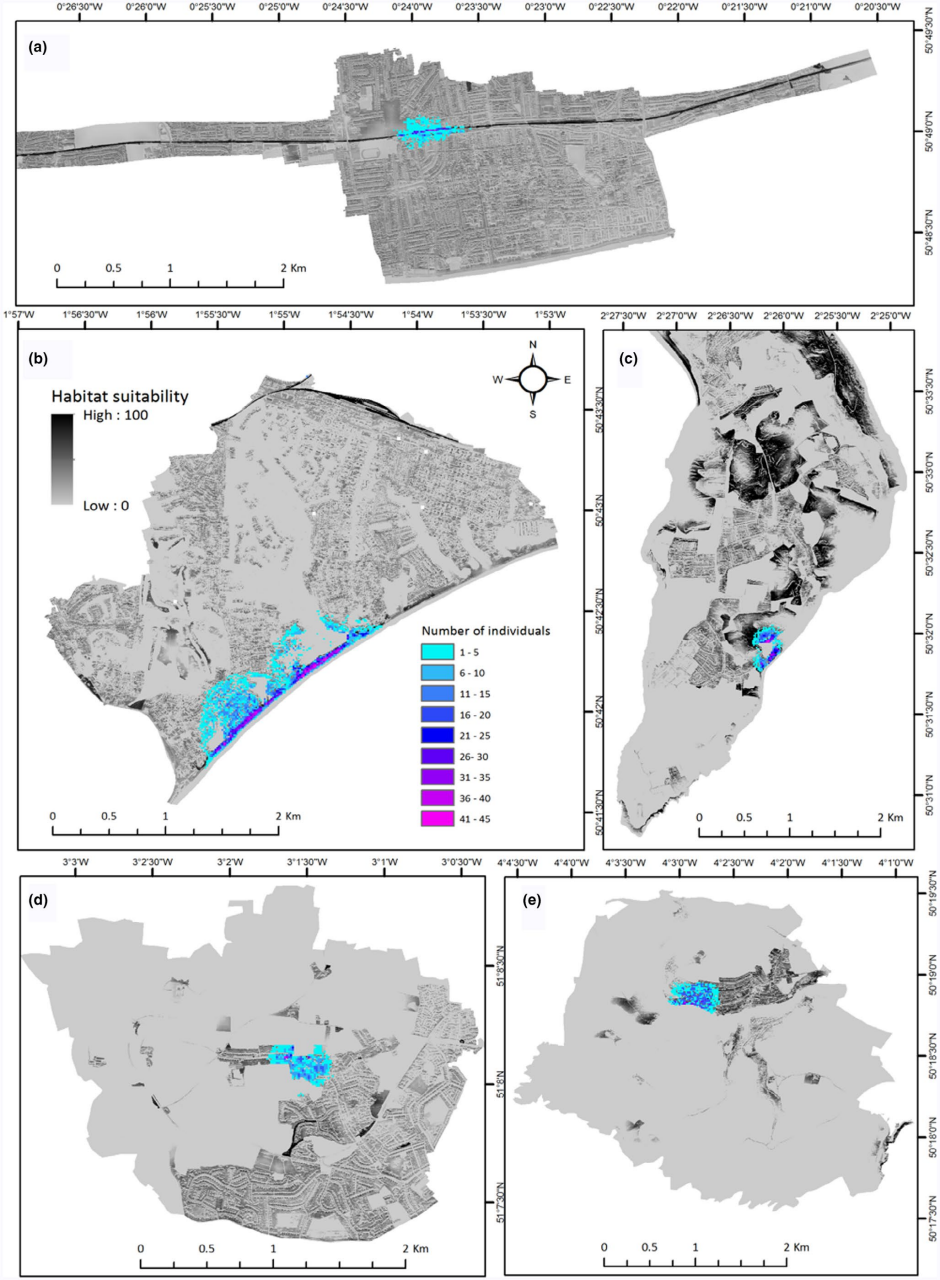
MaxEnt outputs showing local extentand configuration of suitable habitat for *Podarcis muralis* populations in the UK. Order demonstrates the range of variance in patch fragmentation, patch isolation, and linear features of suitable habitat across local landscapes: (a) West Worthing, (b) Bournemouth (including Boscombe and Canford populations), (c) Portland, (d) Wembdon, and (e) Newton Ferrers. Outputs from RangeShifter models are overlain, indicating patterns of population range expansion and lizard density per occupied 225 m^2^ cell projected from year of introduction to 2040

The model fit to the local study areas had an average test AUC of 0.88 (*SD* = 0.01) over the 10 areas and 50 replicated runs. The most important variable to the model was “habitat type,” which made the highest relative contribution to the model (66%). Ten habitat classes out of 44 stood out as being influential to increased suitability for *P. muralis*; bare ground (1), residential garden (2), dense scrub (5), scattered scrub (6), rail track (17), road (18), introduced shrub (22), dry dwarf shrub (25), hard cliff (28), and quarry (37) (Figure 3). Spring insolation had the second‐highest percent contribution to the model (16%), where the amount of spring solar insolation had a positive influence on relative suitability (Figure 3). Relative suitability was also greater closer to buildings, railtrack, and roads. The response to NDVI is one of increasing suitability with an increase in vegetation from bare ground, followed by a rapid negative response past NDVI = 0.5. Maps indicating configuration of suitable habitat within local landscapes are presented in Figure 4 and Figure S4 Appendix S2.

### 4.3 Individual‐based models results

**FIGURE 5:**
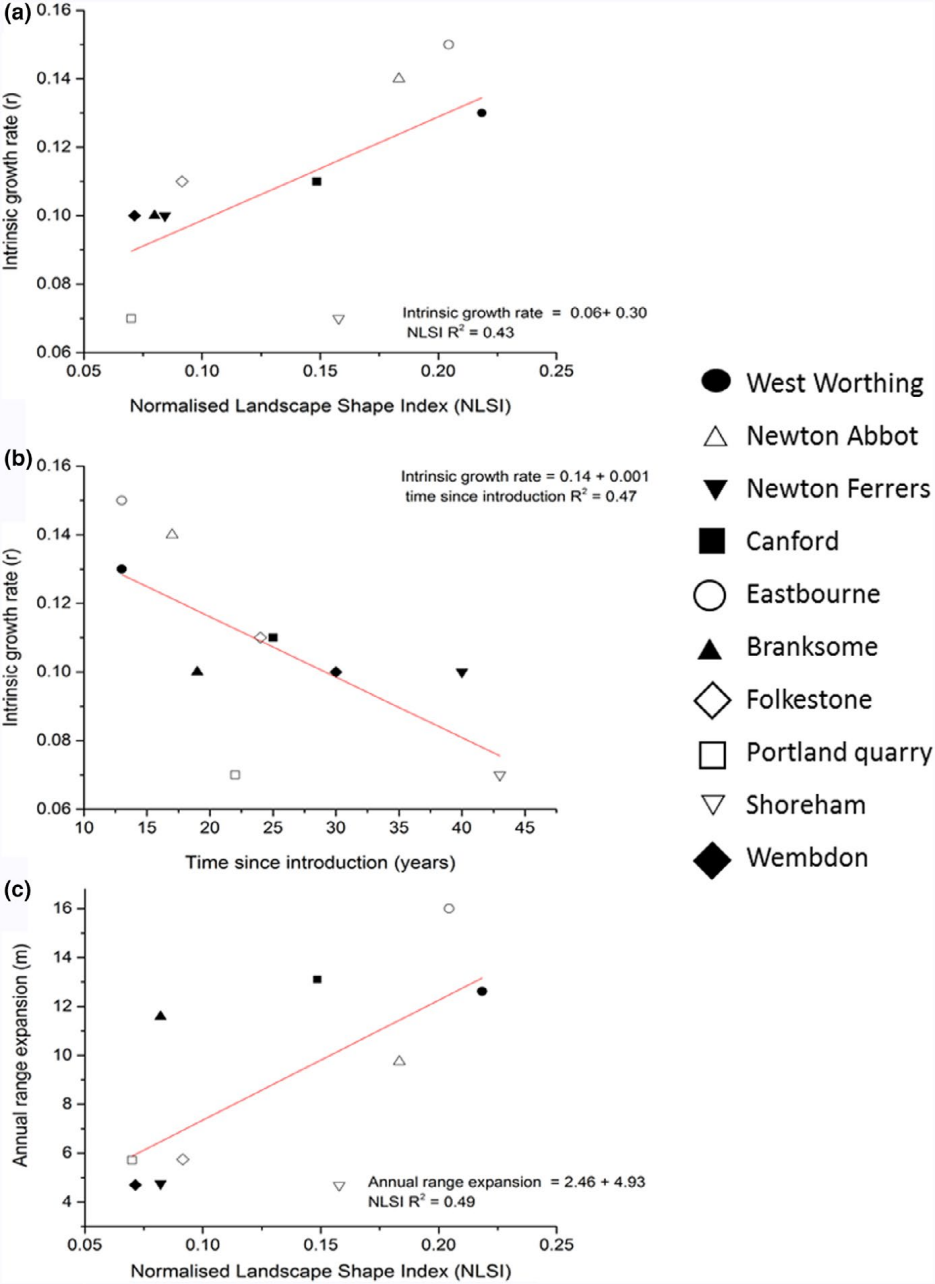
Relationship between growth rate (*r*) and (a) aggregation of suitable habitat (NLSI), time since introduction (b), relationship between NLSI and annual range expansion, and (c) in non‐native population of *Podarcis muralis* in the UK

Patterns of range expansion from time since introduction to 2040, as determined by population dynamics and local landscape character, are presented in Figure 4 and Figure S4 Appendix S2. Growth curves for the 10 study populations are also presented in supplementary information (Figure S5 Appendix S3). Growth rates ranged from 0.07 (Shoreham) to 0.15 (Eastbourne) (Table S3). Growth rate (*r*) was positively related to the NLSI (*F*_(1,9)_ = 8.39, *p* = 0.02, *R* ^2^ = 51.13), and negatively related to time since introduction (*F*_(1,9)_ = 5.80, *p* = 0.04, *R* ^2^ = 42.22) (Figure 5a,b). Branksome and Canford—two populations on the Bournemouth coast—had the highest carrying capacity (10,443 and 10,315 individuals, respectively). Eastbourne had the lowest carrying capacity (1,447) (Table S3). A positive relationship between habitat quality and carrying capacity (*F*_(1,9)_ = 6.22, *p* = 0.03, *R* ^2^ = 43.74) was the only relationship observed between this growth parameter and the explanatory variables (Table 2). Annual range expansion was best explained by combined increases in NLSI and habitat quality (*F*_(2, 9)_ = 29.65, *p* < 0.001, *R* ^2^ = 89.44) (Figure 5c), although habitat quality was not a significant predictor of annual dispersal distance on its own (*F*_(1,9)_ = 1.21, *p* = 0.34, *R* ^2^ = 13.14). Greatest annual range expansion was predicted for the Eastbourne population (16 m), while the Shoreham, Wembdon, and Newton Ferrers populations had similar low expansion of ~4 m per year. Connectance between suitable habitat patches had no relationship with any of the dependent variables.

**TABLE 2:** Summary of separate stepwise regression analysis showing significant variables predicting growth rate (*r*), carrying capacity (*K*), and annual range expansion of introduced *Podarcis muralis* populations in the UK (*N* = 10)

	Model 1		Model 2	
	*Β*	*p*	*β*	*p*
Constant (Annual range expansion)	2.47		−8.07	
NLSI	49.41	0.02	67.78	<0.01
Habitat quality	—	—	0.27	<0.01
R^2^		49.02		89.64
Constant (Intrinsic growth rate)	0.06		0.10	
NLSI	0.32	0.02	0.22	0.07
Time since introduction	—	—	−0.01	0.10
R^2^		51.18		67.27
Constant (Carrying capacity)	−1163.95	0.68		
Habitat quality	218.82	0.03		
R^2^		0.43		

## 5 DISCUSSION

The predicted suitable climate for *P. muralis* in the UK is continuous along the southeast coast, the entire south coast through to the south coast of Wales, extending northward to a latitude of ~52°N—a latitudinal range most likely to reflect maritime climatic conditions found in the species’ native origins. This northern limit to suitable conditions is in keeping with climate matching being an important limiting factor in determining establishment success and range expansion of introduced species, particularly significant for reptiles (Bomford et al. 2009; Mahoney et al. 2015; Pysek et al. 2010). Our climate suitability model output is similar to a previous SDM for *P. muralis*, which also highlighted suitable conditions in the UK up to ~53°N, (Wirga & Majtyka, 2015), despite a differing suite of climatic variables and species presence data informing models.

Our models show that amount of spring radiance, maximum winter temperature, and number of winter ice days were the most informative variables in predicting habitat suitability (where both native and allochthonous records were considered). The hibernation period is short in *P. muralis,* and individuals are often active in mid‐winter during sunny mild spells, even in the northern extremes of their range, making them vulnerable to sudden or prolonged freezing (Claussen et al. 1990). Measurements of critical thermal minimum temperature in an introduced population of *Podarcis sicula* have been shown to be above temperatures likely experienced by some non‐native populations in winter, suggesting individuals may need to find urban thermal retreats to survive winter conditions, or hibernate at a depth below soil freezing to survive (Burke et al. 2002; Liwanag et al. 2018). Interestingly, our UK only model accurately predicted the Greater London Urban Area as having relatively high habitat suitability, likely arising from matching to thermal characteristics associated with the “urban heat island” (UHI) effect (Trajer et al. 2014; Villalobos‐Jimenez & Hassall, 2017). There are historic records of small *P. muralis* populations persisting in this area (Langham, 2019; Langton et al. 2011), and since we did not include these records in the input for the model (due to no recent confirmed sightings and no accurate location data), the predicted suitability in this area gives credence to the validity of the model and the theory of UHI in built environments facilitating overwintering for the species. Dependence on human structures to survive winter temperatures in northern extremes has been suspected for introduced populations of Mediterranean gecko (*Hemidactylus turcicus*) (Locey & Stone, 2006). Microclimatic conditions close to human habitations may have also facilitated establishment of Argentine ant (*Linepithema humilein*) in areas with otherwise unsuitable climate (Roura‐Pascual et al. 2011). Such environments may, however, also act to shield populations from selective pressures that might lead to adaptive physiological responses that could facilitate more rapid diffusion and expansion across wider areas (Hulbert et al. 2020).

Our fine‐scale modeling of habitat suitability provides a detailed insight into local landscape structure and spatial pattern of available suitable habitat. The contribution of habitat classification and spring solar insolation to the model, and particularly the unimodal response observed toward vegetation cover (NDVI), is indicative of the species’ affinities to disturbed habitats that provide resource for refugia (thermal and safety), egg deposition sites, and basking sites necessary for heliothermic temperature regulation (Bertram, 2004; Gherghel et al. 2009). It is possible that although we took great effort to assign habitat type in as much detail as practical, generalizations made during the construction of the habitat classification layer could possibly lead to overestimation of the extent of suitable habitat (e.g., not all habitat classed as residential garden would, in reality, be suitable to *P. muralis*). However, the combined effect of the NDVI variable would go some way to enhance fine‐scale delineation between suitable and unsuitable habitat type.

The relative importance of railway line and introduced shrub habitat in the model can be explained by the number of presence records associated with those habitats in relation to the relative scarcity of those habitats in the landscape. Habitat associated with railway lines provides important habitat for *P. muralis*, facilitating both natural dispersal and accidental human movement of animals (CovaciuMarkov et al. 2006; Gherghel et al. 2009; Kühnis & Schmocker, 2008; Strugariu et al. 2008). Dispersal of the introduced *P. muralis* population in Ohio, Cincinnati, has been reported to be more rapid along the continuous hospitable terrain of rail embankments compared with the relatively slow spread through highly fragmented residential and commercial areas (Hedeen & Hedeen, 1999). Although our simulations of the West Worthing (trackside) population (see Appendix S1) did have relatively higher dispersal distance than most other populations, the pattern of spread did not indicate extensive natural dispersal along the railway, despite the core population being centered on, and around, disused sidings, and associated habitat. Instead, the simulated dispersal pattern is one of predominantly radial diffusion out into adjacent residential and commercial areas, where, although highly fragmented, the habitat was of suitable quality to facilitate this pattern of spread. Linear corridors may, therefore, only become important to natural dispersal when adjacent habitat is of low quality, or is less preferred, as is the case of invasive cane toads (*Rhinella marina*) selecting to use open roads for dispersal through less favorable vegetated habitat (Brown et al. 2006). The presence of other continuous, linear habitat features in our landscape models also increased rates of annual range expansion (e.g., vegetated cliff faces at Branksome and Canford; sea front garden along the promenade at Eastbourne), but this is likely a result of there being restrictions to radial dispersal as suitable habitat is bordered by inferior inland habitat and the shore line. Our findings are congruent with the theory that corridors may be most effective when they actively influence, direct, and channel dispersal rather than simply provide additional suitable habitat (Andrew & Ustin, 2010).

Growth curves derived from our predictive models suggest all the populations studied may be in the early stages of exponential growth, and have demonstrated (or are demonstrating) a lag before the onset of appreciable population growth that is often associated with such a growth trajectory (Sakai et al. 2001). The negative correlation we found between intrinsic growth rate and time since introduction, is to be expected as a function of logistic growth, where the longer‐established populations approach local carrying capacity and density dependence constrains growth (Sibly & Hone, 2002). Increasing densities in urban areas which present transport pathways may lead to an increase long‐distance dispersal events and secondary introductions. A similar scenario has been described in the spread dynamics of invasive Pallas's squirrel (*Callosciurus erythraeus*), where a constant increase in the appearance of new populations occurred after a two‐decade lag, and could be explained by increased vector activity (intentional translocations) as the population size at the initial introduction foci increased—causing a potentially exponential increase of translocation events when translocated populations start acting as a source themselves (Guichón et al., 2015).

Our models concur, however, that natural dispersal of *P. muralis* from points of introductions in the UK is likely to be slow (Foster, 2015), with annual population range expansion of between 5 and16 meters. Spread distances were particularly small for populations in areas of relatively continuous suitable habitat which allows for radial dispersal into suitable neighboring habitat with limited search effort (i.e., rural villages with interconnected gardens, quarries) (Baguette et al. 2013; Bonte et al. 2012). In such instances, it would appear that populations with limited opportunities/need for long‐distance dispersal are increasing their numbers locally, but will be limited for establishing a population over a large area (Lustig et al. 2017). Increasing disaggregation of suitable habitat had a joint positive influence on dispersal rate and growth rate in our models. We found this to be most apparent for the urban population of West Worthing, highlighting how the species’ ability to exploit areas of human disturbance may facilitate overall invasion success (Marvier et al. 2004). Increasing abundance of discrete local patches of suitable habitat may provide opportunity for individuals to disperse more widely in the landscape, thus releasing density‐dependent constraints on population growth that would be in effect when suitable habitat is more aggregated and compact. This pattern is in line with the theories of a percolation threshold, where invasive spread may occur most rapidly and extensively above a threshold level of disturbance (i.e., amount of habitat fragmentation) (With, 2002). In addition, we found functional connectedness of suitable habitat patches had no relation to any of the growth parameters or rate of spread, indicating that localized habitat fragments are acting as stepping stones to dispersal (Alharbi & Petrovskii, 2019). Similar effects of landscape heterogeneity on range expansion of invasive species have been observed in introduced populations of whistling frog (*Eleutherodactylus johnstonei*) (Ernst et al. 2011), Eurasian collared dove (*Streptopelia decaocto*) (Ingenloff et al. 2017), and invasive weeds (Bergelson et al. 1993).

## 6 CONCLUSIONS

Extensive nationwide range expansion through secondary introduction is likely to be restricted by currently unsuitable climate beyond southern regions of the UK. However, exponential growth of local populations in habitats providing transport pathways (e.g., movement of aggregates, timber, plants, and general public) is likely to increase opportunities for long‐distance dispersal and regional expansion.

Despite the fundamental physiological importance of sun exposure to diurnal reptiles, to the best of our knowledge, our models are the first to incorporate estimates of solar insolation into models of habitat suitability at this fine‐scale (but see Garcia‐Porta et al. (2019) for consideration of solar radiation as a variable in driving macroecological and macroevolutionary patterns in lizards). Our models demonstrate the inclusion of the variable at this scale, and indeed our entire approach to developing a fine‐scale SDM could be very useful in other applications relating to ectotherm ecology (e.g., in developing habitat suitability indices, directing habitat management, and guiding survey effort for rare/cryptic species). Furthermore, while the use of SDMs and IBMs have become widely used to further understanding of mechanisms driving invasion dynamics (Fraser et al. 2015; Kadoya & Washitani, 2010; Suzuki‐Ohno et al. 2017), the benefits of incorporating spatially explicit individual‐based models into management plans for the control of invasive species have only recently been recognized (Day et al. 2018). In this regard, our models provide a best estimate for future expansion of *P. muralis* (under current climate conditions) at both the UK national and local scale, providing essential information (i.e., dispersal patterns, key habitat, current and projected population sizes) on which management decisions could be made. It is important to note that forecasts of climate warming may increase suitable area for range expansion and local carrying capacity. As our models show UK populations may be approaching an end to an inherent lag phase, there is an argument to suggest that a timely precautionary intervention may be justified to halt some populations before an abrupt end to the lag phase occurs.

## CONFLICT OF INTEREST

The authors declare no conflict of interest.

## AUTHOR CONTRIBUTIONS

**Robert Williams:** Conceptualization (lead); data curation (lead); formal analysis (lead); funding acquisition (lead); investigation (lead); methodology (lead); project administration (lead); validation (lead); visualization (lead); writing – original draft (lead); writing – review and editing (lead). **Alison Dunn:** Conceptualization (supporting); methodology (supporting); supervision (equal); validation (supporting); writing – review and editing (equal). **Lily Mendes da Costa:** Data curation (equal); formal analysis (equal); investigation (equal); methodology (equal); writing – review and editing (equal). **Chris Hassall:** Conceptualization (supporting); formal analysis (supporting); funding acquisition (equal); investigation (supporting); methodology (supporting); project administration (supporting); supervision (equal); validation (supporting); visualization (supporting); writing – review and editing (equal).

## Supporting information

Appendix S1

Appendix S2

Appendix S3

## Data Availability

All spatial data including presence and background samples with climate/environment data (for local and national‐scale MaxEnt models), and input/output files involved in development and analysis of Rangeshifter simulations are openly available from the NERC Environmental Information Data Center https://doi.org/10.5285/8ae3f9ef‐9a75‐4237‐afbd‐e01abe02e75b (Williams et al., 2021).
