# Supplementary material for: Climate and habitat configuration limit range expansion and patterns of dispersal in a non‐native lizard
Source: Ecol Evol. 2021 Feb 22;11(7):3332–46. doi: 10.1002/ece3.7284 (PMC8019037; doi:10.1002/ece3.7284)
Supplement: Supplementary file 1 — Appendix S1 [file ECE3-11-3332-s002.docx]

Climate and habitat configuration limit range expansion and patterns of dispersal in a non-native lizard

***Appendix S1***

Here, we provide detailed methodologies for the gathering of *P. muralis* location data, details on methods and workflow used in preparing distribution models, and results from spatial analysis of patch aggregation in FRAGSAT for 10 study sites in the South of England.

*Visual surveys*

Surveys took place between 07.30 and 18.00 on days with at least periods of sun, as this weather is considered most productive for lizard surveys (Gent & Gibson, 1998). Sites were walked by 1-4 surveyors paying particular attention to habitat features that provided opportunities for basking and refuge, as lizards aggregate around such features. Locations of lizards were recorded on handheld GPS (Garmin *etrex* 10™) to within ± 1 metre accuracy, or manually recorded by annotating aerial photographs using the Aerial Roam service at https://digimap.edina.ac.uk. Lizard locations collected from GPS were overlain onto aerial images and visually adjusted for precision. As wall lizards are abundant in localised areas and very conspicuous wherever they are present, it was feasible to identify the current extent of most populations by simply walking the area until lizards were no longer encountered.

Specific attention was given to assessing the extent of *P. muralis* presence along railway habitat at West Worthing, Sussex (50.818° N, 0.390° W) during a five-week period in June-July 2018. The railway acted as a linear transect, along which 21 visual survey points and 11 artificial refugia locations were set, providing survey coverage of a transect ~ 9.5km long running from Angmering station to East Worthing station (Fig S1 ). The location of these survey points was determined by limitations to access and therefore it was not possible for the survey effort to be evenly distributed along the transect. Direct access to the railway track was granted by Network Rail, which provided the opportunity for visual surveys and placement of 80 artificial refugia in trackside habitat. Three or four visual survey points were visited per day and searched for 20 minutes at each site. The visual survey points requiring Network Rail access were surveyed four times each, whereas the visual survey points that were accessible to the public were surveyed five times each. Artificial refugia were left undisturbed for one week prior to beginning surveys, to allow them to ‘bed down’. Although it is recommended to leave the refugia to establish for several weeks (Gent & Gibson, 1998) this was not possible due to time constraints. Refugia on public land were surveyed once per week for five weeks, and those on land owned by Network Rail were surveyed once per week for three weeks due to the constraints to access

*Community engagement*

Surveyors took opportunities to engage with the local public during site visits to gain further information into the extent of local lizard populations. When new information was provided, surveyors would extend their search accordingly. Door to door canvasing (at suburban/urban sites) was employed in 2017-2018 to confirm absence of lizards from residential gardens beyond the limits of the population extent observed through visual surveys. Home visits were conducted to investigate presence of lizards within 200m of known locations through random sampling of households. During canvassing, householders’ were either spoken to directly and shown images of wall lizards for identification purposes and asked if they had lizards on their property or seen them locally, or a freepost postcard (coded with street and house number) showing clear pictures of male and female wall lizards was posted through the door asking the same questions and requesting details of where lizards had been seen.


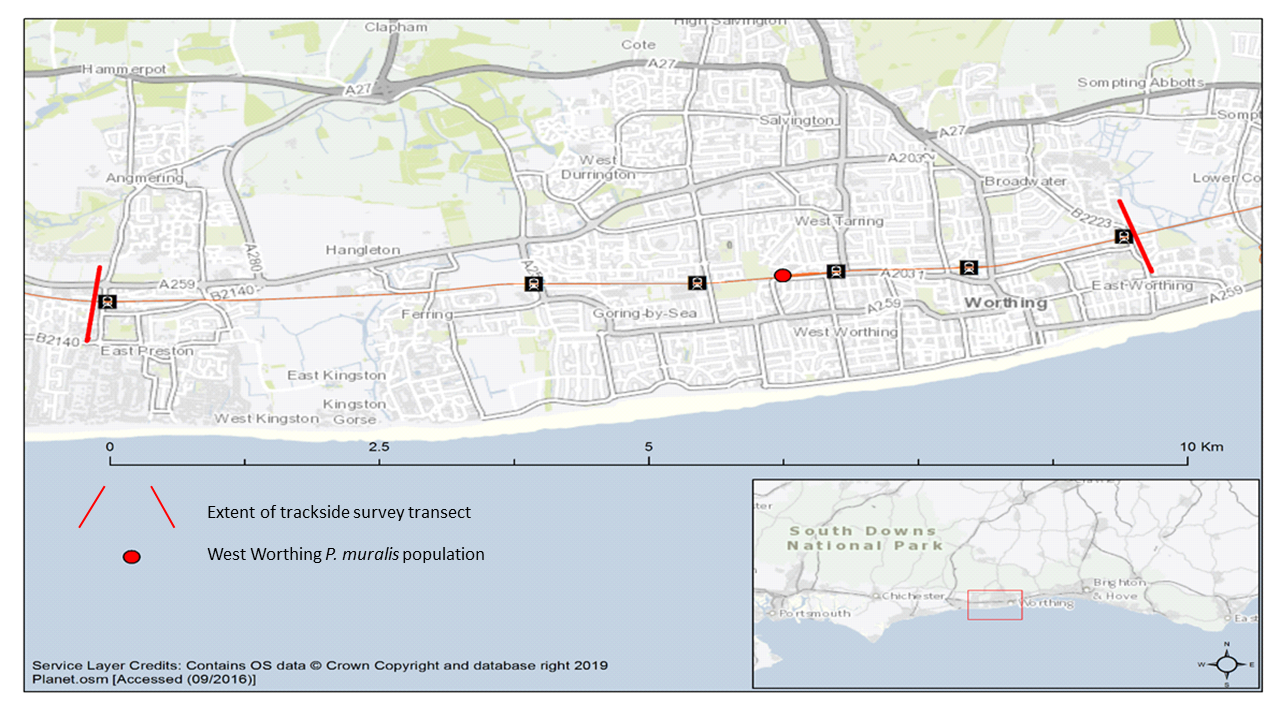
Fig. S1 Extent of 2018 linear survey transect for *P. muralis* along rail track at Worthing, West Sussex, UK

Five hundred postcards were delivered, with a return of 76 (15%). Of these returns, 52 reported confirmed lizard locations. In addition to requesting the return of the postcards regardless of response (confirmation of lizard absence was welcomed), postcards also invited recipients to record their sightings via an online Participatory GIS (PPGIS) hosted through map-me.org (Huck, Whyatt, & Coulton, 2014) where they were given instruction on how to record their sightings on a satellite image of the local area (bit.ly/lizarduk). The PPGIS not only records sightings as locations that can be visualised in GIS software, but also allows researchers to ask questions of participants, the responses to which are stored as attributes linked to these spatial points. In this instance, we asked participants questions that would aid in assessing the veracity of the sightings recorded. Specifically, we asked, 1) Where was the lizard (e.g., on a garden wall, on patio steps)? Such supporting information could describe behaviour indicative of wall lizards, and 2) How confident on a scale of 1-5 (5 being very confident) are you in the accuracy of your identification of wall lizard? Spatial points were reviewed periodically throughout 2017-2018 by overlaying point features onto aerial photographs. Records were validated based on their location, photographic evidence (when provided), and proximity to known populations, in conjunction with question responses attributed to the spatial data. Records were then either rejected outright or retained for confirmation through a site visit. Any points extending the known spatial extent of populations were ground-truthed through additional site visits and widened visual survey in the vicinity of the new sighting records. 114 users engaged with the online portal, of which, 76 generated credible sighting records.

*Press release*

Wall lizard sightings were also generated through a 2017 press release in local news outlets covering the wider distribution of wall lizards in the UK, and where possible, parish magazines covering the 23 study populations. These releases provided information on the wall lizard introduction, pictures to aid identification, and invitation for readers to record sightings at bit.ly/lizarduk or to contact the lead researcher with information directly.

*MaxEnt setup*

To prevent model over-fitting and assist in the interpretation of outputs we minimised model complexity by restricting the functional form of transformations to linear features and increased the regularisation multiplier to two (Merow, Smith, & Silander, 2013; Syfert, Smith, & Coomes, 2013; Merow et al., 2014). All other settings were kept at default values (Phillips & Dudik, 2008). A jackknife test was used in MaxEnt for assessing the effects of individual environmental variables on the prediction accuracy. Area under the receiver operating characteristic curve (test AUC) was used to evaluate models, where test AUC is as a measure of the model’s ability to accurately predict the habitat suitability of a random sample of presence locations that were not used to develop the model (Elith, Kearney, & Phillips, 2010).

For local scale modelling, we supplied presence and background points in the form of SWD (sample with data) files. The model was then projected onto our 10 study sites.

Table S1 Parameters of *P. muralis* biology as used in Rangeshifter simulations, and sources informing input

| Sexual model | Explicit mating system, |  |
| --- | --- | --- |
| Proportion of males | 0.5 | (Vogrin, 1998), this study |
| Max age | 10 | (Kolenda et al., 2020 and references therein) |
| Harem size | 3 | (Oppliger, Degen, John-Alder, & Bouteiller-Reuter, 2007) |
| Stage structure | 3 |  |
| Max fecundity | 12 | (Ji & Brana, 2000; Michaelides et al., 2016) |
| Breeding chance | 1 | (Pellitteri-Rosa et al., 2012) |
| Density dependent fecundity | yes | (Massot, Clobert, Pilorge, Lecomte, & Barbault, 1992) |
| Probability survival to adulthood (yr 2) | Male 22% female 28% | (Barbault & Mou, 1988; While et al., 2015) |
| Strength of density dependence 1/b (i.e., slope of the negative exponential function which governs density dependence in fecundity) | 1200 |  |
| Transfer mechanism | Stochastic movement simulator | Cost layer derived from local maxent models |
| Emmigration probability | Density dependent (sex and stage dependent)  Juvenile male *D0*  0.9; female 0.8 (α = 10, β =0)  Stage 1 male *D0* 0.8 ; female 0.7 (α = 10, β =1)  Stage 2 male *D0* 0.2; female 0.1 (α = 10, β = 1) | (Vignoli, Vuerich, & Bologna, 2012) |
| Step mortality | 0.01-0.05 |  |
| Perceptual range | 1 |  |
| Directional persistance | 5 |  |
| Settlement (both sexes) density dependent | Find a suitable cell + density dependent. Density dependence slope -10 infl. point 0.7 |  |
| Min steps | 0 |  |
| Max steps | 420 |  |
| Max steps per year | 40 (600m yr) | (Schulte, Veith, Mingo, Modica, & Hochkirch, 2013) |

Table S2 Results from spatial analysis of patch aggregation in FRAGSAT for 10 study sites in the South of England. NLSI = Normalised Landscape Shape Index (0-1)

| **Site** | **nlsi** | **Connectance** % |
| --- | --- | --- |
| Newton Abbot | 0.1833 | 2.6944 |
| Branksome | 0.0821 | 3.0582 |
| Canford | 0.1485 | 6.1859 |
| Eastbourne | 0.2044 | 3.9141 |
| Newton Ferrers | 0.0815 | 9.1361 |
| Folkestone | 0.0915 | 3.9756 |
| Portland | 0.07 | 9.0986 |
| Shoreham | 0.1578 | 7.5979 |
| Wembdon | 0.0714 | 15.3439 |
| West Worthing | 0.2184 | 1.7496 |

**
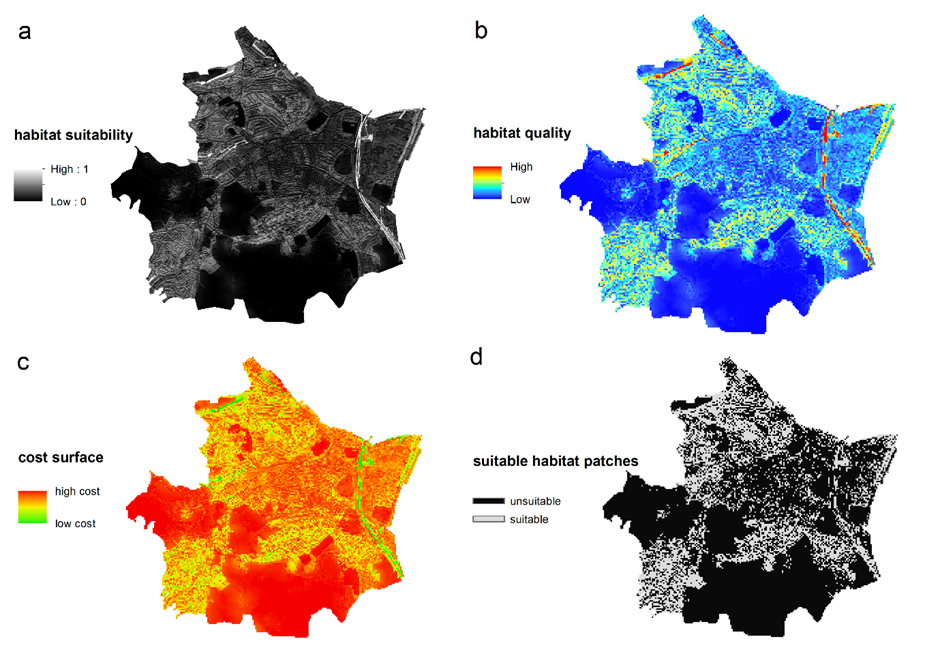
**

Figure S2 Illustration of workflow from a) MaxEnt logistic output of habitat suitability, b) Rescaled habitat quality used as input for RangeShifter, c) Cost surface layer used as input for RangeShifter, through to d) Binary map of suitable and unsuitable habitat used as input for Fragstats analysis

**References**

Barbault, R., & Mou, Y. P. (1988). Population-dynamics of the common wall lizard, *Podarcis-Muralis*, in Southwestern France. *Herpetologica, 44*(1), 38-47.

Elith, J., Kearney, M., & Phillips, S. J. (2010). The art of modelling range-shifting species. *Methods in Ecology and Evolution, 1*(4), 330-342. doi:10.1111/j.2041-210X.2010.00036.x

Gent, A., & Gibson, S. (1998). *Herpetofauna Workers' Manual*. Retrieved from Peterborough:

Huck, J. J., Whyatt, J. D., & Coulton, P. (2014). Spraycan: A PPGIS for capturing imprecise notions of place. *Applied Geography, 55*, 229-237. doi:10.1016/j.apgeog.2014.09.007

Ji, X. A., & Brana, F. (2000). Among clutch variation in reproductive output and egg size in the wall lizard (*Podarcis muralis*) from a lowland population of northern Spain. *Journal of Herpetology, 34*(1), 54-60. doi:Doi 10.2307/1565238

Kolenda, K., Skawiński, T., Majtyka, T., Majtyka, M., Kuśmierek, N., Starzecka, A., & Jablonski, D. (2020). Biology and origin of isolated north-easternmost populations of the common wall lizard, Podarcis muralis %J Amphibia-Reptilia. *Amphibia-Reptilia*, 1-15. doi:<https://doi.org/10.1163/15685381-bja10011>

Massot, M., Clobert, J., Pilorge, T., Lecomte, J., & Barbault, R. (1992). Density dependence in the common lizard - demographic consequences of a density manipulation. *Ecology, 73*(5), 1742-1756. doi:Doi 10.2307/1940026

Merow, C., Smith, M. J., Edwards, T. C., Guisan, A., McMahon, S. M., Normand, S., . . . Elith, J. (2014). What do we gain from simplicity versus complexity in species distribution models? *Ecography, 37*(12), 1267-1281. doi:10.1111/ecog.00845

Merow, C., Smith, M. J., & Silander, J. A. (2013). A practical guide to MaxEnt for modeling species' distributions: what it does, and why inputs and settings matter. *Ecography, 36*(10), 1058-1069. doi:10.1111/j.1600-0587.2013.07872.x

Michaelides, S. N., While, G. M., Zajac, N., Aubret, F., Calsbeek, B., Sacchi, R., . . . Uller, T. (2016). Loss of genetic diversity and increased embryonic mortality in non-native lizard populations. *Mol Ecol, 25*(17), 4113-4125. doi:10.1111/mec.13755

Oppliger, A., Degen, L., John-Alder, H.-B., & Bouteiller-Reuter, C. (2007). Promiscuity and high level of multiple paternity in common wall lizards (*Podarcis muralis*): data from microsatellite markers. *28*(2), 301. doi:<https://doi.org/10.1163/156853807780202477>

Pellitteri-Rosa, D., Sacchi, R., Pupin, F., Bellati, A., Cocca, W., Gentilli, A., . . . Fasola, M. (2012). Testing the ability to store sperm: an experimental manipulation of mating opportunities in the common wall lizard, *Podarcis muralis*. *Acta Herpetologica, 7*(1), 111-118.

Phillips, S. J., & Dudik, M. (2008). Modeling of species distributions with Maxent: new extensions and a comprehensive evaluation. *Ecography, 31*(2), 161-175. doi:10.1111/j.0906-7590.2008.5203.x

Schulte, U., Veith, M., Mingo, V., Modica, C., & Hochkirch, A. (2013). Strong genetic differentiation due to multiple founder events during a recent range expansion of an introduced wall lizard population. *Biological Invasions, 15*(12), 2639-2649. doi:10.1007/s10530-013-0480-5

Syfert, M. M., Smith, M. J., & Coomes, D. A. (2013). The effects of sampling bias and model complexity on the predictive performance of MaxEnt species distribution models. *PLoS One, 8*(2). doi:ARTN e55158

10.1371/journal.pone.0055158

Vignoli, L., Vuerich, V., & Bologna, M. A. (2012). Experimental study of dispersal behaviour in a wall lizard species (*Podarcis sicula*) (Sauria Lacertidae). *Ethology Ecology & Evolution, 24*(3), 244-256. doi:10.1080/03949370.2011.643922

Vogrin, N. (1998). Demography of a Slovenian population of the Wall Lizard *Podarcis muralis muralis* (LAURENTI, 1768). *Herpetozoa, 11*(1), 13-17.

While, G. M., Williamson, J., Prescott, G., Horvathova, T., Fresnillo, B., Beeton, N. J., . . . Uller, T. (2015). Adaptive responses to cool climate promotes persistence of a non-native lizard. *Proceedings of the Royal Society B-Biological Sciences, 282*(1803). doi:Artn 20142638

10.1098/Rspb.2014.2638
