# Supplementary material for: Climate and habitat configuration limit range expansion and patterns of dispersal in a non‐native lizard
Source: Ecol Evol. 2021 Feb 22;11(7):3332–46. doi: 10.1002/ece3.7284 (PMC8019037; doi:10.1002/ece3.7284)
Supplement: Supplementary file 2 — Appendix S2 [file ECE3-11-3332-s003.docx]

Climate and habitat configuration limit range expansion and patterns of dispersal in a non-native lizard

***Appendix S2***

Here, we provide a graphic illustrating MaxEnt outputs showing response curves for *P. muralis* habitat suitability to 10 environmental variables as modelled in MaxEnt at the UK national scale (Fig S2). Three models are presented, each using different presence data (Native range, native + UK, UK only). In Figure S3, we present local extent and configuration of suitable habitat for *P. muralis* populations in the UK. Outputs from RangeShifter models are overlain, indicating patterns of population dispersal projected from year of introduction to 2040 and number of lizards per occupied 225m^2^cell


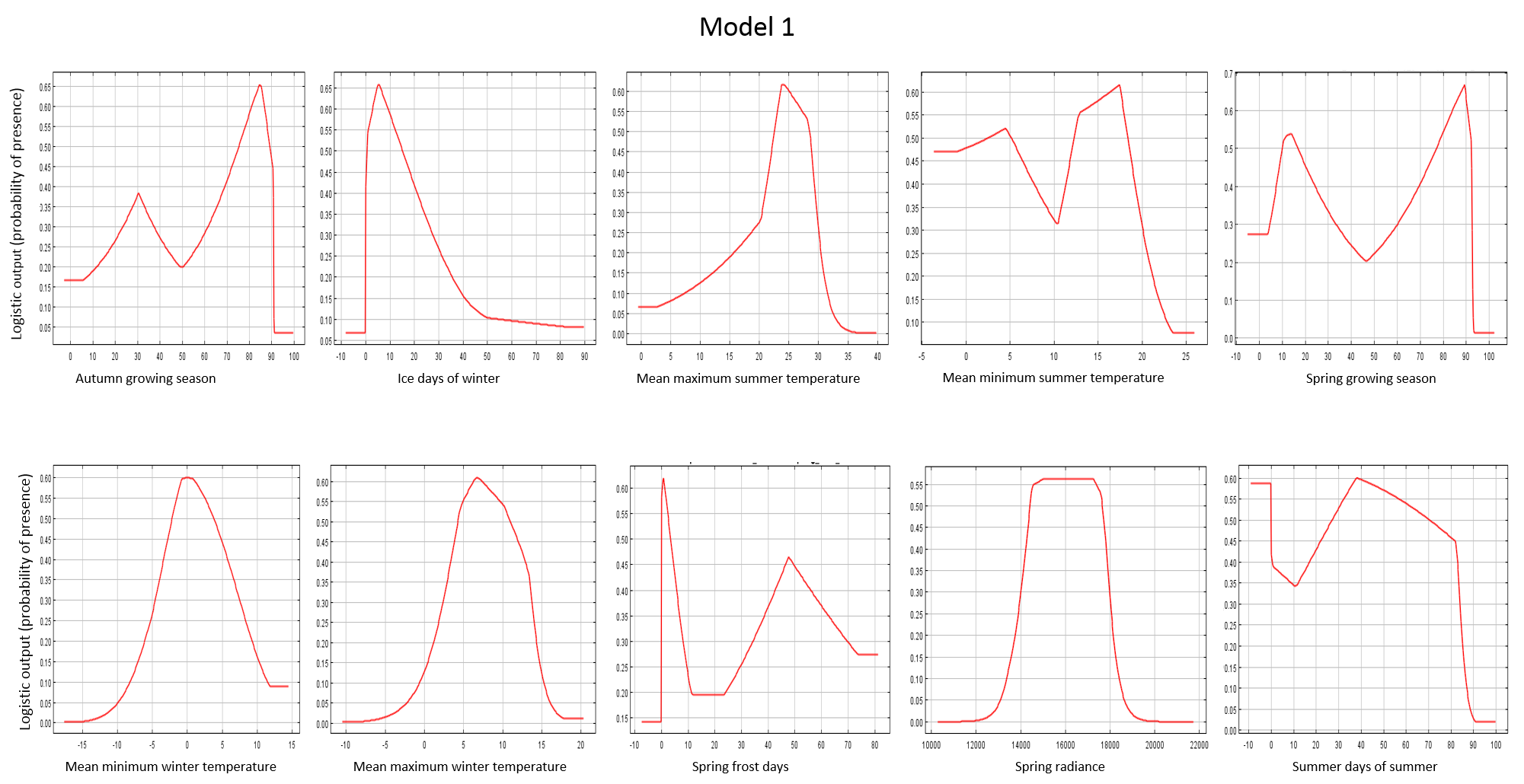


Logistic output (suitability)


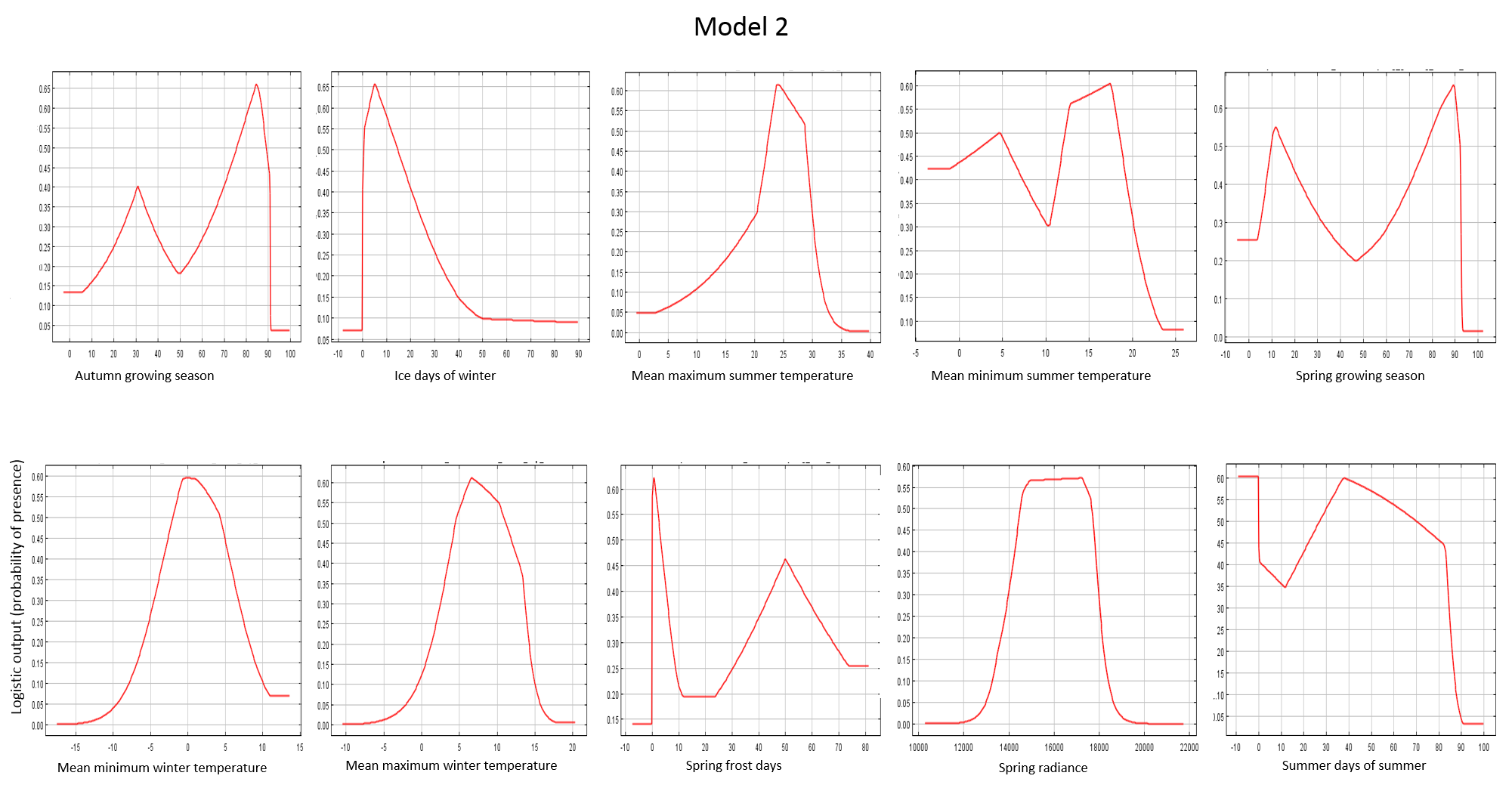


Logistic output (suitability)


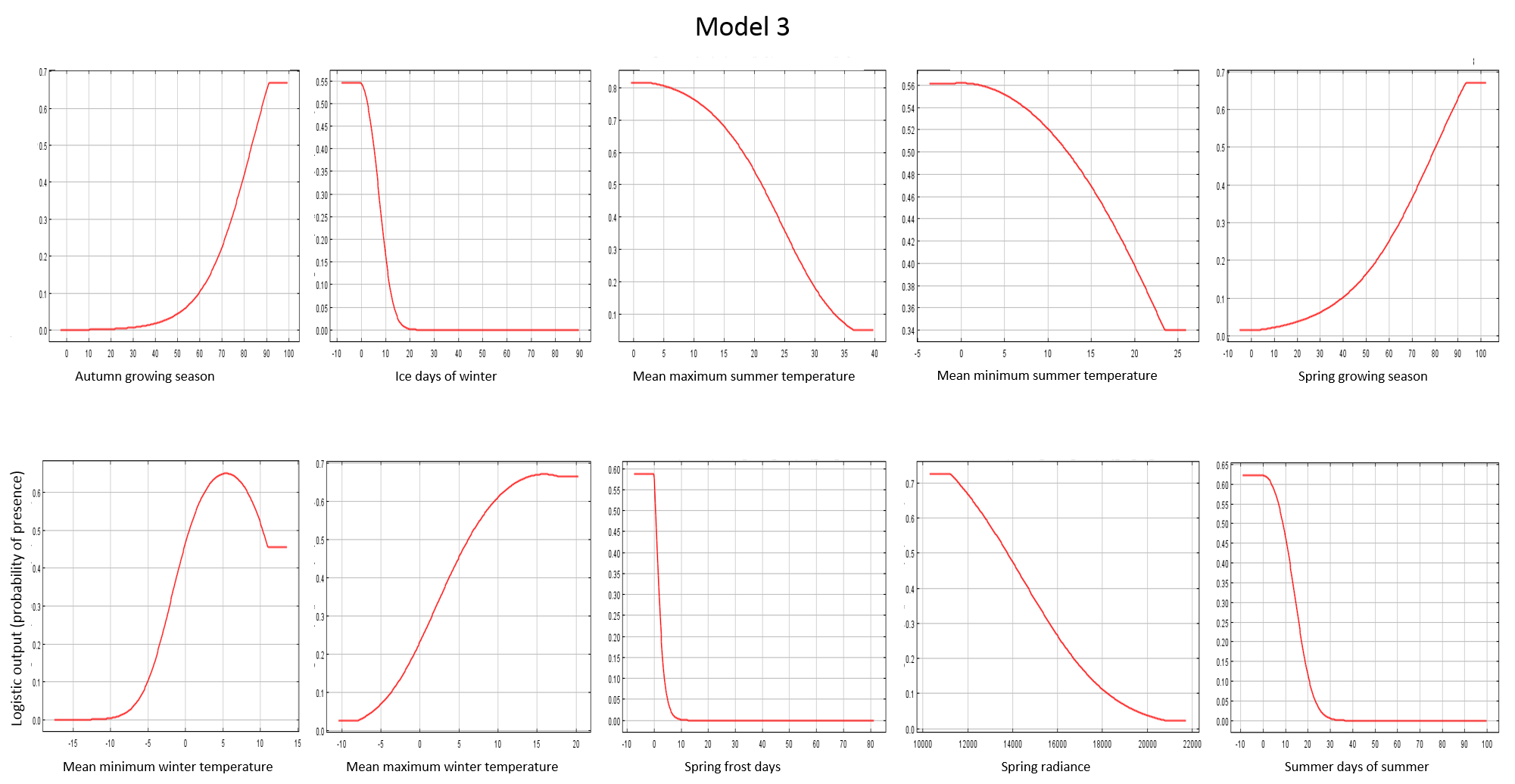


Logistic output (suitability)

Figure S2. Response curves (suitability) for *P. muralis* to 10 environmental variables as modelled in MaxEnt at the UK national scale. Model is informed by presence records from the native range only. Model 2 is informed by presence records from the native range and the allochthonous UK records. Model 3 is informed by the UK records only. Each individual plot represents a Maxent model created using only the corresponding variable


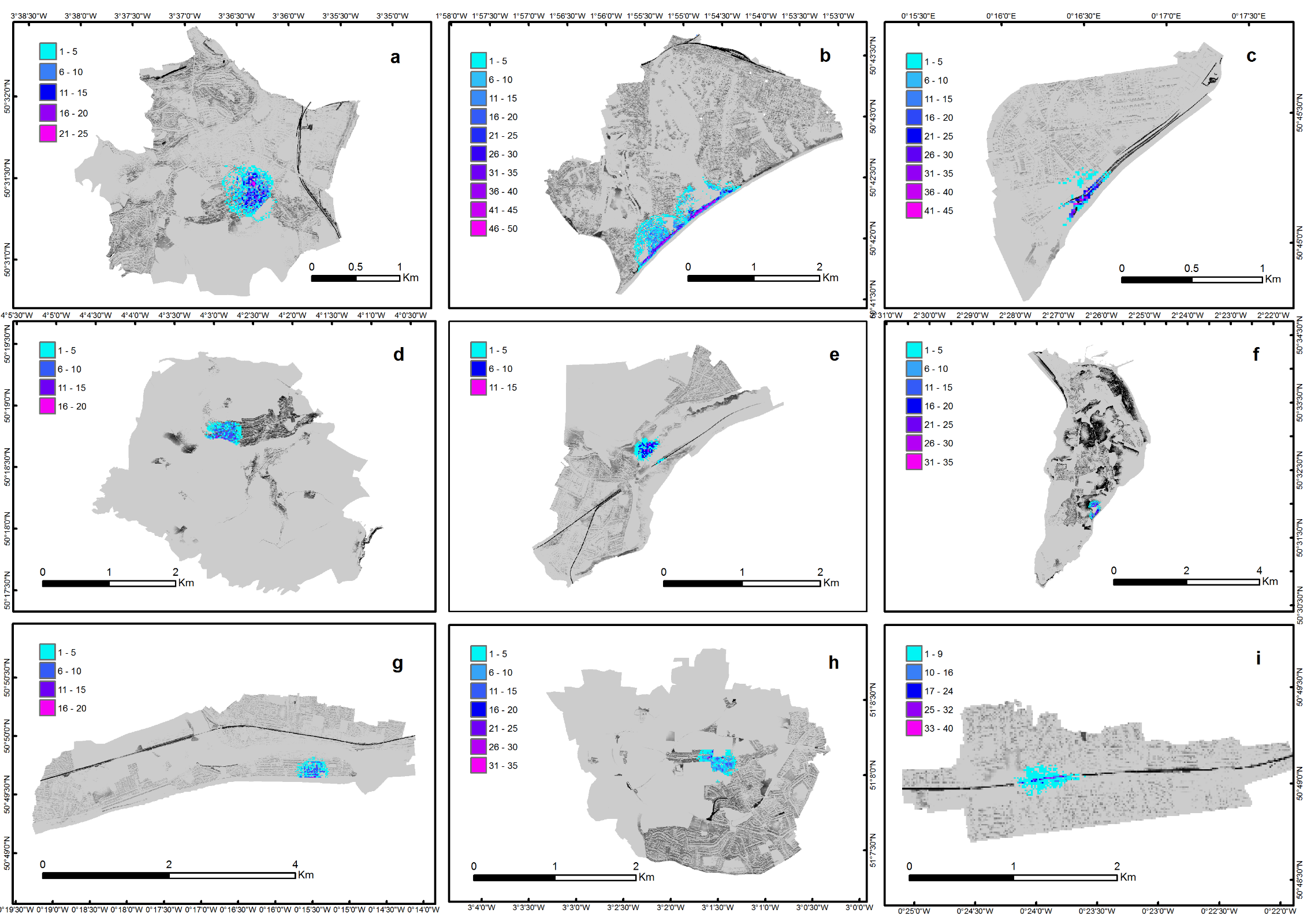


Figure S3. MaxEnt outputs showing local extent and configuration of suitable habitat for *P. muralis* populations in the UK: a) Newton Abbot, b) Bournemouth (including Boscombe and Canford populations), c) Eastbourne, d) Newton Ferrers, e) Folkestone, f) Portland, g) Shoreham, h) Wembdon, i) West Worthing. Outputs from RangeShifter models are overlain, indicating patterns of range expansion projected from year of introduction to 2040 and number of lizards per occupied 225m^2^cell
