# Supplementary material for: Climate and habitat configuration limit range expansion and patterns of dispersal in a non‐native lizard
Source: Ecol Evol. 2021 Feb 22;11(7):3332–46. doi: 10.1002/ece3.7284 (PMC8019037; doi:10.1002/ece3.7284)
Supplement: Supplementary file 3 — Appendix S3 [file ECE3-11-3332-s001.docx]

Climate and habitat configuration limit range expansion and patterns of dispersal in a non-native lizard

***Appendix S3***

Here, we provide estimated growth parameters and predicted growth curves for ten introduced population of *P. muralis* in the UK, projected from year of introduction to 2040.

Table S3 Estimated parameters of intrinsic growth rate (r) and carrying capacity (K) that summarise growth curves for 10 populations of Podarcis muralis in the UK, as calculated in the R package Growthcurver

| Site | Intrinsic growth rate (*r*) ± se | Carrying capacity (K) ± se |
| --- | --- | --- |
| Newton Abbot | 0.148 ± 0.001 | 7623 ± 155 |
| Branksome | 0.106 ± 0.005 | 10443 ± 1007 |
| Canford | 0.116 ± 0.004 | 10315 ± 247 |
| Eastbourne | 0.158 ± 0.004 | 1447 ± 26.8 |
| Newton Ferrers | 0.102 ± 0.001 | 3964 ± 50.8 |
| Folkestone | 0.116 ± 0.002 | 1960 ± 36.9 |
| Portland | 0.075 ± 0.003 | 8132 ± 1814 |
| Shoreham | 0.078 ± 0.002 | 2122 ± 86.6 |
| Wembdon | 0.109 ± 0.001 | 3609 ± 105 |
| West Worthing | 0.131 ± 0.005 | 3867 ± 146 |

Figure S5. Predicted growth curves for ten introduced population of *P. muralis* in the UK. Data points represent yearly mean population size across 50 RangeShifter replicates. Vertical line = estimated population size at time of surveying. Note that the y-axes differ markedly among populations, indicating large variation in the population sizes.
